# Diseases of the Antrum

**Published:** 1890-06

**Authors:** E. Catt

**Affiliations:** U. of M.


### Diseases of the Antrum.

BY E. CATT, U. OF M.

LOCATION.—The antrum of Highmore, known as early as the time of Galen, has been variously described by all modern anatomists. Its location, surrounded as it is by important structures, renders the study of its pathological conditions an important one for surgeons.

Placed in the superior maxillary bone, it is compared by Holden to a triangular pyramid, the base toward the nose, the apex to the maler bone; its roof forms the floor of the crbit, its floor is the alveolar process and its anterior and posterior walls form portions of the facial and zygomatic surfaces. It communicates with the middle meatus of the nasal fossa by an aperture through which, in diseased conditions, purulent matter is frequently discharged, forming a diagnostic sign.

The extreme thinness of its walls gives an easy explanation of the fact that a tumor, originating in this cavity, will, by pushing up the orbital plate, displace the eye ball; or, projecting inward, enter the nasal fossa; or, making its way backward pass into the zygomatic fossa and downward into the mouth.

Mr. Cattlin, in a paper read before the Odont. Soc'y in 1858, draws attention to the fact that the antrum is sometimes divided into two or more compartments by thin, bony plates which may run either vertically or horizontally. A knowledge of these irregular formations will assist in the diagnosis and treatment of the diseases of this cavity. For instance, suppose an operation for tapping be made; unless such a condition as the above be recognized, one compartment will be evacuated whilst the other will still retain its secretions; or, the instrument impinging upon the bony septum, it may be mistaken for an osseous growth.

CAUSES.—The principal causes of disease of the antrum are, 1, diseased teeth; 2, affections of the mucous membrane; 3, foreign bodies. Of the three causes enumerated, the first, or diseased teeth, is by far the most active.

The roots of the first and second molars are so intimately connected with the floor of the antrum that when they become carious the thin plate of bone covering them often becomes affected and disease is set up in the cavity. Acute inflammation of the antrum is often caused by pus from a dental abscess.

A common cause of inflammation of the mucous membrane of the antrum is catarrh of the Schneiderian membrane of the nose, which, by continuity of tissue, causes engorgement of the lining membrane of the antrum. Mercury may be a cause through the periosteal inflammation it induces in the alveoli.

Peas, buttons, &c., are sometimes thrust into the nares by children of an experimental turn of mind; these, of course, are a source of offence and cause irritation of the mucous membrane which may extend to the antrum.

Abscess.—The most common cause of abscess of the antrum is diseased teeth; but it may have a traumatic origin, the history of many cases commencing with the receipt of a violent blow. It has been excited by the entrance of foreign bodies from the mouth after the extraction of a tooth communicating with the cavity.

Signs and Symptoms.—A dull, deep-seated pain shooting up the face to the forehead, swelling and tenderness of the cheek, a slight chill, denotes the formation of pus, which, if the disease is sufficiently advanced, may be seen issuing from that side of the nose. An abscess progresses rapidly with signs of acute inflammation, thus differing from a tumor, which is slow in growth and presents no signs of activity.

If the opening into the nares be closed by congestion of the mucous membrane, the pus, unable to find an exit, may push up the floor of the orbit, displacing the eye or cause a bulging of the cheek, or, pressing downward, displace the palate, and even more serious results have followed. Dr. Mair of Madras, recording a case in the *Edin. Med. Jour.* for May 1866, of a gentleman in whom suppuration of the antrum was followed by death in sixteen days from suppuration within the cranium.

Treatment.—The two points to be kept in view are *free drainage and cleanliness.* To secure the first, it is generally advisable to extract one of the molar teeth, and through the socket drill a free opening into the cavity; then wash out well with tepid distilled water and plug the opening with cotton wool. Sometimes this is sufficient; if, however, progress is not satisfactory, inject a detergent and astringent solution. 1 part of iodine to 2 of alcohol is recommended. Mild solutions of permanganate of potash and sulphate of zinc are also useful.

Antracele—Or dropsy of the antrum is only another name for mucous engorgement. The cause is an irritant, producing a mild inflammation of the mucous membrane which gives rise to a watery secretion. Reflected periodontal inflammation from a diseased tooth is not an unfrequent cause.

Garretson states that the most common cause of engorgement of the antrum is simple catarrh of the Schneiderian membrane, which, by continuity of tissue, affects the mucous lining of that cavity.

Signs and Symptoms.—There is a gradual swelling of the upper jaw with a feeling of pressure, but no pain, and the general health is not impaired. The mucous membrane being now thickened and puffy, the opening communicating with the nasal fossa will be closed and the secretion, accumulating within the cavity, presses upon its walls, which, under the constant pressure, become gradually attenuated, until a slight pressure made upon them produces a crackling sound, as of parchment. Sometimes when absorption has taken place, fluctuation can be felt.

Treatment.—The fluid may be evacuated by several methods; 1, catheterism may be effected by passing a curved probe into the middle meatus of the nasal fossa, where the aperture of the antrum may be reopened, but as this opening is above the floor of the sinus, this treatment is not as a rule effectual. 2, a hole may be drilled within the mouth at the upper part of the alveolar process, at the point of attachment of the muscles to the jaw and as this should pierce the floor of the antrum, is much more likely to be effective. 3, a molar may be extracted and entrance made through the socket. In all cases where a carious molar exists, this method should be chosen.

Free drainage having been obtained, syringe well with sterilized water and inject a solution of iodine and alcohol, one part of the former to two of the latter. This, however, being somewhat painful treatment, it is recommended that it be preceded by an injection of a 4 per cent. solution of cocaine.

Where the secretion, being of an acrid character, has given rise to degenerating ulcers, an injection of a solution of sulphate of zinc will generally be satisfactory in its results. For general treatment, give tonics, tinc. ferri. chlor. M. x-xv gtts diluted after meals, is a good one.

Cysts.—A cyst may be defined as a cavity having a distinct wall and containing fluid. Cysts are more commonly found originating from the roots of teeth, but frequently involve the antrum.

The external appearance of one of these cysts so much resembles that of a solid growth, that they may readily be mistaken for an osseous tumor. For a positive diagnosis therefore, the exploring needle should always be used. Small cysts do sometimes form in the antrum and are explained by M. Giraldes as being due to the dilatation of the glanular follicles of the mucous membrane.

Treatment.—Make a free opening into the antrum, pass in the finger and search for any growth or foreign body that may be lodged within and mop out with tinct. iodi. Dr. Martin then recommends packing the cavity with cotton covered with powdered boracic acid.

Tumors of The Antrum.—These, as in other parts of the body, may be divided into malignant and non-malignant, and have the same characteristics. Fortunately, however, those belonging to the non-malignant variety are most common in the upper jaw.

Fibrous Tumors.—These are the growths most common to the upper jaw. They grow slowly and are non-malignant. They usually arise from the periosteum, or, more rarely, the endosteum. The cause of one of these growths may generally be traced to a blow, or to the irritation produced by diseased roots of teeth. This irritation, causing determination of blood to the part, produces hypertrophy and excessive proliferation of the tissue cells.

Diagnosis.—They grow slowly but may attain an enormous size. In the museum of the College of Surgeons, London, is a specimen weighing 12 pounds. They may remain stationary for years and give no pain, until by gradual increase in size they press upon adjacent parts. They are hard and firm and do not affect the general health of the patient.

Treatment.—In many situations these tumors may be simply let alone, until by reason of their gradual increase in size they press upon surrounding tissues, causing pain and arrest of nutrition; but in all cases where they are found in the antrum, the neck or the angle of the jaw, Erichsen recommends their extirpation.

Osteomata—Consists of a stroma of fibrous tissue containing bone cells only; they are non-malignant and have the same characteristics as the fibromata, growing slowly and without pain. They are hard and firm and frequently cease to grow after a time, remaining passive for the rest of the patient's life.

Mr. Heath cites a remarkable case of osseous tumor, in which, after twenty-three years, the growth originating in the anterior wall of the antrum and having displaced the eye ball, separated spontaneously from the face by ulceration round its base, the process of separation occupying six years and being finally accomplished without pain or hæmorrhage.

Enchondromatous and myeloid tumors are other forms of non-malignant growths, which, however, rarely occur in this situation.

Carcinoma—Or cancer is the general name given to tumors of a malignant nature. These growths consist of a stroma which is essentially the tissue of the part in which the tumor is developing, containing epithelical cells, which cells are not confined by a capsule, but rapidly proliferating, invade and incorporate the adjacent tissues.

There are two varieties of cancer, the encephaloid and the Scirrhus, and of these the encephaloid is the most common in the maxillary bones. It is characterised by all the signs of malignancy, grows rapidly and soon breaks down, ulcerating and forming fungus masses from which frequent hæmorrhages occur.

These are often troublesome, as vessels incorporated into the growth lose their power of contraction and retraction. Garretson states that the case is exceptional where the patient survives over two years.

Heath is of opinion that in most cases of encephaloma of the upper jaw, the disease originates in the antrum, and if, as in a case of Mr. Liston's, the superior maxillary bone is removed, whilst the disease is confined to that bone, all may be well, but in the majority of cases its growth is so rapid, the oral, nasal and orbital cavities are so early involved that nothing can be done. There being no line of demarcation, the surgeon is utterly at a loss where to introduce the knife, and so, whilst doing his best to palliate the condition of his patient can give him no hope of cure.

APHRODISIAC effects from cocaine have been reported by several observers, and the possibility of producing strong sexual excitement by the use of a small quantity of the drug should be borne in mind. With male patients this effect is of but little consequence, but where the physician is alone with a female patient the consequences may be embarrassing if nothing worse. A Philadelphia physician reports giving a hypodermic injection of a few drops of a ten per cent. solution of cocaine to a woman from whose face he proposed to remove a small tumor. The erotic excitement that followed led the patient to behave in a most unseemly way although her usual behavior was modest and becoming. A St. Paul dentist reports a similar experience, his patient making an indecent exposure of the person while under the influence of a small injection of cocaine. Dentists should indeed be particularly on their guard, since they use cocaine so often in filling and extracting teeth, and are so frequently alone with their patients.—*Northwestern Lancet.*

DEXTRINE MUCILAGE.—Four hundred parts of dextrine are stirred into 400 parts of water; then add 200 parts more of water, 20 parts of glucose and 10 parts of aluminum sulphate; heat the mixture to 195° F., when it will become thin and transparent.

